# 4-(2,3-Dimethyl­anilino)pent-3-en-2-one

**DOI:** 10.1107/S1600536812038779

**Published:** 2012-09-15

**Authors:** Gertruida J. S. Venter, Gideon Steyl, Andreas Roodt

**Affiliations:** a Department of Chemistry, University of the Free State, PO Box 339, Bloemfontein, 9300, South Africa

## Abstract

In the title compound, C_13_H_17_NO, the dihedral angle between the aryl ring and the amino­acryl­aldehyde mean plane [N—C=C—C=O; maximum deviation = 0.0144 (9) Å] is 53.43 (4)°. There is an intra­molecular N—H⋯O hydrogen bond involving the amine and carbonyl groups. In the crystal, mol­ecules are linked by C—H⋯O hydrogen bonds, forming chains propagating along [001].

## Related literature

For background to the synthesis of the title compound, see: Shaheen *et al.* (2006 ▶); Venter *et al.* (2010 ▶). For applications of rhodium compounds containing bidentate ligand systems, see: Pyżuk *et al.* (1993 ▶); Tan *et al.* (2008 ▶); Xia *et al.* (2008 ▶). For related rhodium enamino­ketonato complexes, see: Brink *et al.* (2010 ▶); Damoense *et al.* (1994 ▶); Roodt & Steyn (2000 ▶); Venter *et al.* (2009*a* ▶,*b* ▶; 2012 ▶).
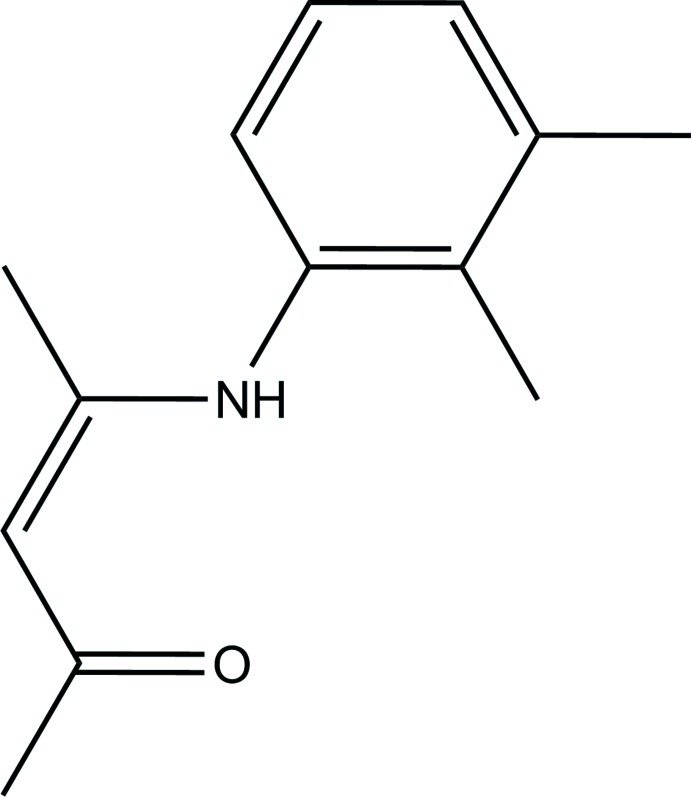


## Experimental

### 

#### Crystal data

C_13_H_17_NO
*M* *_r_* = 203.28
Monoclinic, 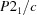

*a* = 7.526 (3) Å
*b* = 12.450 (5) Å
*c* = 12.040 (4) Å
β = 90.243 (4)°
*V* = 1128.1 (7) Å^3^
*Z* = 4
Mo *K*α radiation
μ = 0.08 mm^−1^
*T* = 100 K
0.18 × 0.16 × 0.08 mm

#### Data collection

Bruker APEXII CCD area-detector diffractometer
Absorption correction: multi-scan (*SADABS*; Bruker, 2004 ▶) *T*_min_ = 0.987, *T*_max_ = 0.994
20265 measured reflections
2817 independent reflections
2528 reflections with *I* > 2σ(*I*)
*R*_int_ = 0.024

#### Refinement

*R*[*F* ^2^ > 2σ(*F* ^2^)] = 0.038
*wR*(*F* ^2^) = 0.103
*S* = 1.05
2817 reflections
144 parameters
H atoms treated by a mixture of independent and constrained refinement
Δρ_max_ = 0.33 e Å^−3^
Δρ_min_ = −0.24 e Å^−3^

### 

Data collection: *APEX2* (Bruker, 2005 ▶); cell refinement: *SAINT-Plus* (Bruker, 2004 ▶); data reduction: *SAINT-Plus*; program(s) used to solve structure: *SIR92* (Altomare *et al.*, 1992) ▶; program(s) used to refine structure: *SHELXL97* (Sheldrick, 2008 ▶); molecular graphics: *DIAMOND* (Brandenburg & Putz, 2005 ▶); software used to prepare material for publication: *WinGX* (Farrugia, 1999 ▶).

## Supplementary Material

Crystal structure: contains datablock(s) global, I. DOI: 10.1107/S1600536812038779/su2465sup1.cif

Structure factors: contains datablock(s) I. DOI: 10.1107/S1600536812038779/su2465Isup2.hkl

Supplementary material file. DOI: 10.1107/S1600536812038779/su2465Isup3.cml

Additional supplementary materials:  crystallographic information; 3D view; checkCIF report

## Figures and Tables

**Table 1:** Hydrogen-bond geometry (Å, °)

*D*—H⋯*A*	*D*—H	H⋯*A*	*D*⋯*A*	*D*—H⋯*A*
N1—H1⋯O1	0.911 (15)	1.869 (15)	2.6348 (13)	140.2 (13)
C1—H1*A*⋯O1^i^	0.98	2.49	3.4599 (15)	173

Symmetry code: (i) 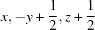.

**Fig. 1.**
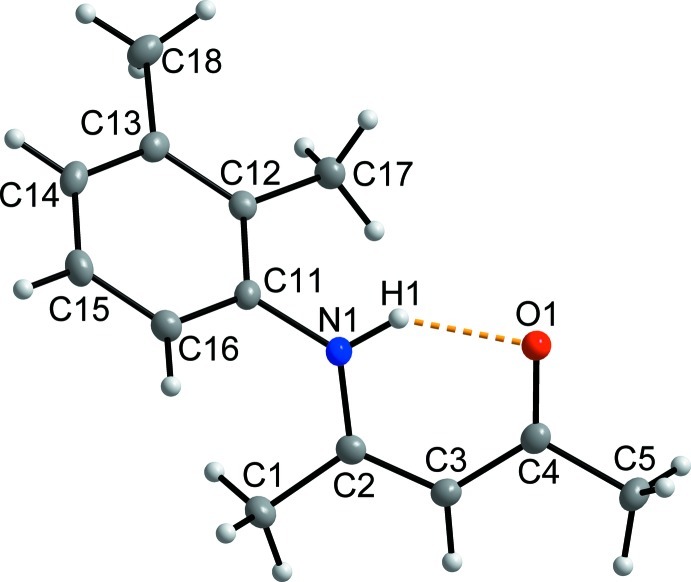
The molecular structure of the title molecule, with the atom numbering. the displacement ellipsoids are drawn at the 50% probability displacement level. The intramolecular N—H···O hydrogen bond is shown as a yellow dashed line (see Table 1 for details).

## supplementary crystallographic information

### Comment

The β-diketone compound AcacH (acetylacetone; or when coordinated acetylacetonato, acac^-^) has been studied extensively, with a multitude of derivatives synthesized to date. One such derivative type, known as enaminoketones, containing N and O atoms as well as an unsaturated C═C bond, and are of interest in various fields including liquid crystals (Pyżuk *et al.*, 1993) and fluorescence studies (Xia *et al.*, 2008). They also have significant application possibilities in medicine (Tan *et al.*, 2008)] and catalysis (Roodt & Steyn, 2000; Brink *et al.*, 2010).

The title compound (Fig. 1) is an enaminoketone derivative of 4-(phenylamino)pent-3-en-2-one (Shaheen *et al.*, 2006). Bond distances in the the title compound differ significantly from those in compounds where the ligand is coordinated to rhodium (Venter *et al.*, 2009**a*,b*; 2012); Damoense *et al.*, 1994), but it share characteristics with other enaminoketones of this type (Venter *et al.*, 2010). The C2–C3 bond distance of 1.3849 (14) Å *versus* the C3–C4 distance of 1.4251 (13) Å indicates an unsaturated bond in the pentenone backbone. Here the intramolecular distance N1···O1 is 2.6348 (13) Å which is considerably less (~ 0.2 Å) than that observed when the ligand is coordinated to rhodium for example (Venter *et al.*, 2009**a*,b*; 2012; Damoense *et al.*, 1994).

The intramolecular N-H···O hydrogen bond that is formed (Fig. 1 and Table 1) enhances the planarity of the aminopentenone moiety. The aminoacrylaldehyde mean plane [N1-C2═C3-C4═O1; maximum deviation = 0.0144 (9) Å] makes a dihedral angle of 53.43 (4)° with the C11-C16 benzene ring. This angle is dependent on the position of the substituents on the aromatic ring. Compounds with substituents in the *ortho* positions result in larger dihedral angles, while smaller angles are found for derivatives with substituents in the *para* position (Venter *et al.*, 2009**a*,b*; 2012).

In the crystal, there are C-H···O hydrogen bonds leading to the formation of chains propagating along [001] (Table 1 and Fig. 2).

### Experimental

The title compound was prepared following the literature procedure (Shaheen *et al.*, 2006; Venter *et al.*, 2010).

### Refinement

The NH H atom was located in a difference Fourier map and freely refined. The H atoms were placed in geometrically idealized positions and constrained to ride on their parent atoms: C—H = 0.95 and 0.98 Å for CH and CH_3_ H atoms, respectively, with *U*_iso_(H) = k × *U*_eq_(C), where k = 1.5 for CH_3_ H atoms and = 1.2 for other H atoms. The methyl groups were generated to fit the difference electron density and the groups were then refined as rigid rotors.

### Crystal data

**Table:**

C_13_H_17_NO	*F*(000) = 440
*M**_r_* = 203.28	*D*_x_ = 1.197 Mg m^−^^3^
Monoclinic, *P*2_1_/*c*	Mo *K*α radiation, λ = 0.71073 Å
Hall symbol: -P 2ybc	Cell parameters from 9978 reflections
*a* = 7.526 (3) Å	θ = 2.7–28.4°
*b* = 12.450 (5) Å	µ = 0.08 mm^−^^1^
*c* = 12.040 (4) Å	*T* = 100 K
β = 90.243 (4)°	Cuboid, colourless
*V* = 1128.1 (7) Å^3^	0.18 × 0.16 × 0.08 mm
*Z* = 4	

### Data collection

**Table:**

Bruker APEXII CCD area-detector diffractometer	2817 independent reflections
Radiation source: fine-focus sealed tube	2528 reflections with *I* > 2σ(*I*)
Graphite monochromator	*R*_int_ = 0.024
phi and ω scans	θ_max_ = 28.4°, θ_min_ = 2.4°
Absorption correction: multi-scan (*SADABS*; Bruker, 2004)	*h* = −9→10
*T*_min_ = 0.987, *T*_max_ = 0.994	*k* = −16→16
20265 measured reflections	*l* = −15→16

### Refinement

**Table:**

Refinement on *F*^2^	Primary atom site location: structure-invariant direct methods
Least-squares matrix: full	Secondary atom site location: difference Fourier map
*R*[*F*^2^ > 2σ(*F*^2^)] = 0.038	Hydrogen site location: inferred from neighbouring sites
*wR*(*F*^2^) = 0.103	H atoms treated by a mixture of independent and constrained refinement
*S* = 1.05	*w* = 1/[σ^2^(*F*_o_^2^) + (0.0535*P*)^2^ + 0.3712*P*] where *P* = (*F*_o_^2^ + 2*F*_c_^2^)/3
2817 reflections	(Δ/σ)_max_ < 0.001
144 parameters	Δρ_max_ = 0.33 e Å^−^^3^
0 restraints	Δρ_min_ = −0.24 e Å^−^^3^

### Special details

**Table:**

Geometry. All s.u.'s (except the s.u. in the dihedral angle between two l.s. planes) are estimated using the full covariance matrix. The cell s.u.'s are taken into account individually in the estimation of s.u.'s in distances, angles and torsion angles; correlations between s.u.'s in cell parameters are only used when they are defined by crystal symmetry. An approximate (isotropic) treatment of cell s.u.'s is used for estimating s.u.'s involving l.s. planes.
Refinement. Refinement of *F*^2^ against ALL reflections. The weighted *R*-factor *wR* and goodness of fit *S* are based on *F*^2^, conventional *R*-factors *R* are based on *F*, with *F* set to zero for negative *F*^2^. The threshold expression of *F*^2^ > 2σ(*F*^2^) is used only for calculating *R*-factors(gt) *etc*. and is not relevant to the choice of reflections for refinement. *R*-factors based on *F*^2^ are statistically about twice as large as those based on *F*, and *R*- factors based on ALL data will be even larger.

### Fractional atomic coordinates and isotropic or equivalent isotropic displacement parameters (Å^2^)

**Table:**

	*x*	*y*	*z*	*U*_iso_*/*U*_eq_	
C1	0.06673 (13)	0.30089 (8)	0.27466 (8)	0.0206 (2)	
H1A	0.1442	0.3069	0.34	0.031*	
H1B	−0.0323	0.2525	0.2913	0.031*	
H1C	0.0202	0.372	0.2552	0.031*	
C2	0.17093 (11)	0.25693 (7)	0.17890 (7)	0.01569 (19)	
C3	0.15832 (12)	0.14919 (7)	0.15135 (7)	0.01678 (19)	
H3	0.083	0.1047	0.1946	0.02*	
C4	0.25242 (12)	0.10152 (7)	0.06148 (7)	0.01660 (19)	
C5	0.23616 (14)	−0.01799 (8)	0.04301 (9)	0.0233 (2)	
H5A	0.1841	−0.0315	−0.0304	0.035*	
H5B	0.1597	−0.0491	0.1003	0.035*	
H5C	0.3542	−0.051	0.0472	0.035*	
C11	0.29955 (12)	0.43578 (7)	0.13435 (7)	0.01577 (19)	
C12	0.27101 (11)	0.50391 (7)	0.04322 (7)	0.01532 (19)	
C13	0.30377 (12)	0.61452 (7)	0.05608 (8)	0.01670 (19)	
C14	0.36216 (12)	0.65366 (8)	0.15862 (8)	0.0192 (2)	
H14	0.385	0.7283	0.167	0.023*	
C15	0.38734 (12)	0.58538 (8)	0.24843 (8)	0.0199 (2)	
H15	0.4246	0.6136	0.318	0.024*	
C16	0.35811 (13)	0.47587 (8)	0.23660 (8)	0.0185 (2)	
H16	0.3777	0.4286	0.2974	0.022*	
C17	0.20554 (13)	0.46041 (8)	−0.06639 (8)	0.0190 (2)	
H17A	0.3054	0.4546	−0.118	0.028*	
H17B	0.1158	0.5091	−0.0974	0.028*	
H17C	0.1529	0.3893	−0.055	0.028*	
C18	0.27215 (14)	0.69111 (8)	−0.03884 (9)	0.0223 (2)	
H18A	0.1441	0.6989	−0.0515	0.034*	
H18B	0.3282	0.6629	−0.1061	0.034*	
H18C	0.3235	0.7613	−0.0206	0.034*	
N1	0.27422 (11)	0.32320 (6)	0.11920 (7)	0.01722 (18)	
O1	0.35012 (9)	0.15398 (5)	−0.00317 (6)	0.01903 (16)	
H1	0.3236 (19)	0.2906 (12)	0.0590 (12)	0.033 (4)*	

### Atomic displacement parameters (Å^2^)

**Table:**

	*U*^11^	*U*^22^	*U*^33^	*U*^12^	*U*^13^	*U*^23^
C1	0.0210 (4)	0.0217 (5)	0.0193 (4)	−0.0008 (4)	0.0040 (3)	−0.0023 (3)
C2	0.0148 (4)	0.0177 (4)	0.0146 (4)	0.0006 (3)	−0.0018 (3)	0.0005 (3)
C3	0.0176 (4)	0.0159 (4)	0.0169 (4)	−0.0007 (3)	0.0001 (3)	0.0017 (3)
C4	0.0167 (4)	0.0155 (4)	0.0176 (4)	0.0002 (3)	−0.0033 (3)	0.0006 (3)
C5	0.0264 (5)	0.0150 (4)	0.0285 (5)	−0.0019 (4)	0.0046 (4)	−0.0016 (4)
C11	0.0155 (4)	0.0138 (4)	0.0181 (4)	0.0003 (3)	0.0018 (3)	−0.0018 (3)
C12	0.0125 (4)	0.0166 (4)	0.0168 (4)	0.0006 (3)	0.0009 (3)	−0.0014 (3)
C13	0.0135 (4)	0.0154 (4)	0.0212 (4)	0.0012 (3)	0.0011 (3)	0.0002 (3)
C14	0.0166 (4)	0.0153 (4)	0.0258 (5)	0.0000 (3)	0.0005 (3)	−0.0041 (3)
C15	0.0183 (4)	0.0221 (5)	0.0192 (4)	0.0002 (3)	−0.0010 (3)	−0.0063 (4)
C16	0.0190 (4)	0.0197 (4)	0.0170 (4)	0.0009 (3)	−0.0003 (3)	−0.0005 (3)
C17	0.0207 (4)	0.0194 (4)	0.0168 (4)	−0.0008 (3)	−0.0012 (3)	−0.0009 (3)
C18	0.0227 (5)	0.0171 (4)	0.0272 (5)	0.0002 (4)	−0.0018 (4)	0.0039 (4)
N1	0.0209 (4)	0.0139 (4)	0.0169 (4)	−0.0001 (3)	0.0031 (3)	−0.0015 (3)
O1	0.0223 (3)	0.0165 (3)	0.0183 (3)	−0.0011 (3)	0.0025 (3)	−0.0004 (2)

### Geometric parameters (Å, º)

**Table:**

C1—C2	1.5005 (13)	C12—C13	1.4074 (14)
C1—H1A	0.98	C12—C17	1.5074 (13)
C1—H1B	0.98	C13—C14	1.3964 (14)
C1—H1C	0.98	C13—C18	1.5066 (14)
C2—N1	1.3441 (12)	C14—C15	1.3878 (14)
C2—C3	1.3849 (14)	C14—H14	0.95
C3—C4	1.4251 (13)	C15—C16	1.3883 (14)
C3—H3	0.95	C15—H15	0.95
C4—O1	1.2562 (12)	C16—H16	0.95
C4—C5	1.5093 (14)	C17—H17A	0.98
C5—H5A	0.98	C17—H17B	0.98
C5—H5B	0.98	C17—H17C	0.98
C5—H5C	0.98	C18—H18A	0.98
C11—C16	1.3979 (13)	C18—H18B	0.98
C11—C12	1.4027 (13)	C18—H18C	0.98
C11—N1	1.4262 (13)	N1—H1	0.911 (15)
			
C2—C1—H1A	109.5	C14—C13—C12	119.54 (8)
C2—C1—H1B	109.5	C14—C13—C18	119.86 (9)
H1A—C1—H1B	109.5	C12—C13—C18	120.58 (9)
C2—C1—H1C	109.5	C15—C14—C13	121.10 (9)
H1A—C1—H1C	109.5	C15—C14—H14	119.4
H1B—C1—H1C	109.5	C13—C14—H14	119.5
N1—C2—C3	120.37 (8)	C14—C15—C16	120.03 (9)
N1—C2—C1	119.50 (8)	C14—C15—H15	120
C3—C2—C1	120.12 (8)	C16—C15—H15	120
C2—C3—C4	123.46 (8)	C15—C16—C11	119.35 (9)
C2—C3—H3	118.3	C15—C16—H16	120.3
C4—C3—H3	118.3	C11—C16—H16	120.3
O1—C4—C3	123.23 (9)	C12—C17—H17A	109.5
O1—C4—C5	117.94 (8)	C12—C17—H17B	109.5
C3—C4—C5	118.82 (8)	H17A—C17—H17B	109.5
C4—C5—H5A	109.5	C12—C17—H17C	109.5
C4—C5—H5B	109.5	H17A—C17—H17C	109.5
H5A—C5—H5B	109.5	H17B—C17—H17C	109.5
C4—C5—H5C	109.5	C13—C18—H18A	109.5
H5A—C5—H5C	109.5	C13—C18—H18B	109.5
H5B—C5—H5C	109.5	H18A—C18—H18B	109.5
C16—C11—C12	121.31 (9)	C13—C18—H18C	109.5
C16—C11—N1	120.33 (8)	H18A—C18—H18C	109.5
C12—C11—N1	118.32 (8)	H18B—C18—H18C	109.5
C11—C12—C13	118.65 (9)	C2—N1—C11	127.75 (8)
C11—C12—C17	121.06 (8)	C2—N1—H1	113.0 (9)
C13—C12—C17	120.29 (8)	C11—N1—H1	119.0 (9)

### Hydrogen-bond geometry (Å, º)

**Table:**

*D*—H···*A*	*D*—H	H···*A*	*D*···*A*	*D*—H···*A*
N1—H1···O1	0.911 (15)	1.869 (15)	2.6348 (13)	140.2 (13)
C1—H1*A*···O1^i^	0.98	2.49	3.4599 (15)	173

Symmetry code: (i) *x*, −*y*+1/2, *z*+1/2.
